# 3-Chloro-4-fluoro­anilinium picrate

**DOI:** 10.1107/S1600536813000718

**Published:** 2013-01-16

**Authors:** Balladka K. Sarojini, Badiadka Narayana, Hemmige S. Yathirajan, Thomas Gerber, Benjamin van Brecht, Richard Betz

**Affiliations:** a P. A. College of Engineering, Department of Chemistry, Nadupadavu, Mangalore 574 153, India; b Mangalore University, Department of Studies in Chemistry, Mangalagangotri 574 199, India; c University of Mysore, Department of Studies in Chemistry, Manasagangotri, Mysore 570 006, India; d Nelson Mandela Metropolitan University, Summerstrand Campus, Department of Chemistry, University Way, Summerstrand, PO Box 77000, Port Elizabeth, 6031, South Africa

## Abstract

In the title picrate salt of a dihalogenated aniline derivative, C_6_H_6_ClF^+^·C_6_H_2_N_3_O_7_^−^, the intra­cyclic C—C—C angles in the picrate anion cover a broad range [111.95 (12)–125.38 (13)°], while those in the aromatic cation span a much narrower range [118.25 (14)–122.33 (13)°]. In the crystal, classical N—H⋯O hydrogen bonds, as well as C—H⋯O contacts, connect the ions into layers parallel to (001).

## Related literature

For related structures, see: Jin *et al.* (2011 ▶); Wang (2011 ▶); Betz *et al.* (2011 ▶); Dutkiewicz *et al.* (2011 ▶); Jasinski *et al.* (2010*a* ▶,*b* ▶, 2011 ▶). For graph-set analysis of hydrogen bonds, see: Etter *et al.* (1990 ▶); Bernstein *et al.* (1995 ▶).
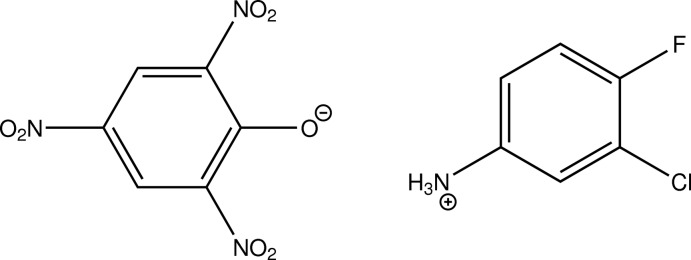


## Experimental

### 

#### Crystal data

C_6_H_6_ClF^+^·C_6_H_2_N_3_O_7_^−^
*M* *_r_* = 374.67
Triclinic, 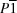

*a* = 4.4054 (2) Å
*b* = 11.9881 (5) Å
*c* = 13.7010 (5) Å
α = 90.057 (1)°
β = 91.803 (1)°
γ = 97.743 (1)°
*V* = 716.62 (5) Å^3^
*Z* = 2
Mo *K*α radiation
μ = 0.33 mm^−1^
*T* = 200 K
0.53 × 0.32 × 0.13 mm

#### Data collection

Bruker APEXII CCD diffractometer
Absorption correction: multi-scan (*SADABS*; Bruker, 2008 ▶) *T*_min_ = 0.907, *T*_max_ = 1.000
12360 measured reflections
3525 independent reflections
2947 reflections with *I* > 2σ(*I*)
*R*_int_ = 0.014

#### Refinement

*R*[*F* ^2^ > 2σ(*F* ^2^)] = 0.037
*wR*(*F* ^2^) = 0.097
*S* = 1.06
3525 reflections
238 parameters
H atoms treated by a mixture of independent and constrained refinement
Δρ_max_ = 0.32 e Å^−3^
Δρ_min_ = −0.27 e Å^−3^

### 

Data collection: *APEX2* (Bruker, 2010 ▶); cell refinement: *SAINT* (Bruker, 2010 ▶); data reduction: *SAINT*; program(s) used to solve structure: *SHELXS97* (Sheldrick, 2008 ▶); program(s) used to refine structure: *SHELXL97* (Sheldrick, 2008 ▶); molecular graphics: *ORTEP-3 for Windows* (Farrugia, 2012 ▶) and *Mercury* (Macrae *et al.*, 2008 ▶); software used to prepare material for publication: *SHELXL97* and *PLATON* (Spek, 2009 ▶).

## Supplementary Material

Crystal structure: contains datablock(s) I, global. DOI: 10.1107/S1600536813000718/bg2490sup1.cif

Supplementary material file. DOI: 10.1107/S1600536813000718/bg2490Isup2.cdx

Structure factors: contains datablock(s) I. DOI: 10.1107/S1600536813000718/bg2490Isup3.hkl

Supplementary material file. DOI: 10.1107/S1600536813000718/bg2490Isup4.cml

Additional supplementary materials:  crystallographic information; 3D view; checkCIF report

## Figures and Tables

**Table 1:** Hydrogen-bond geometry (Å, °)

*D*—H⋯*A*	*D*—H	H⋯*A*	*D*⋯*A*	*D*—H⋯*A*
N4—H41⋯O1^i^	0.91 (2)	1.85 (2)	2.7324 (17)	165 (2)
N4—H42⋯O12^ii^	0.89 (2)	2.40 (2)	3.0599 (18)	131.2 (16)
N4—H42⋯O11^ii^	0.89 (2)	2.60 (2)	3.3425 (17)	141.8 (16)
N4—H43⋯O1	0.96 (2)	1.81 (2)	2.7579 (16)	172.7 (18)
C13—H13⋯O21^iii^	0.95	2.47	3.3152 (19)	148
C26—H26⋯O32	0.95	2.49	3.378 (2)	156

Symmetry codes: (i) 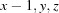; (ii) 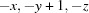; (iii) 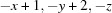.

**Fig. 1.**
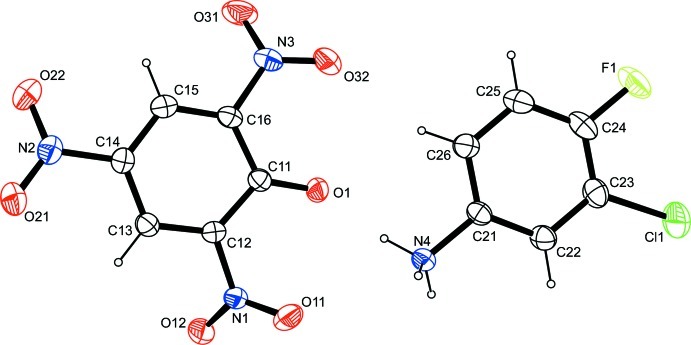
The molecular structure of the title compound, with atom labels and anisotropic displacement ellipsoids (drawn at 50% probability level).

**Fig. 2.**
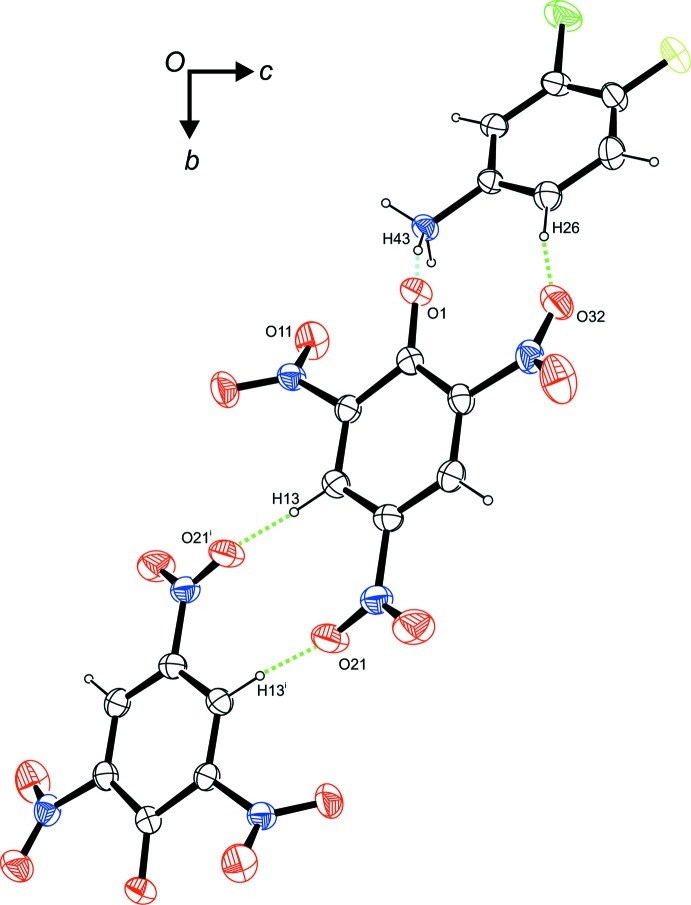
Intermolecular contacts, viewed along [-1 0 0]. For reasons of clarity, only a selection of intermolecular contacts is shown. Blue dashed lines depict classical hydrogen bonds of the N–H···O type, green dashed lines depict C–H···O contacts. Symmetry operator: i -x + 1, -y + 2, -z.

**Fig. 3.**
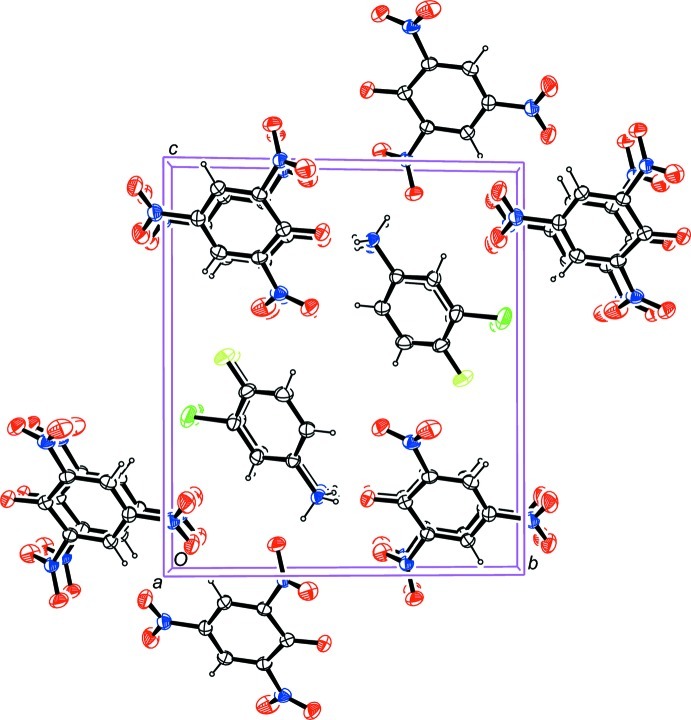
Molecular packing of the title compound, viewed along [-1 0 0] (anisotropic displacement ellipsoids drawn at 50% probability level).

## supplementary crystallographic information

### Comment

2,4,6-Trinitrophenol (picric acid) was and is primarily used to manufacture explosives. I has also found widespread use as an intermediate in the production of dyes. As a strong organic acid, picric acid forms salts with a large variety of *N*-containing organic bases. The crystal structures of some picrates have been reported (Jin *et al.*, 2011; Wang, 2011; Betz *et al.*, 2011; Dutkiewicz *et al.*, 2011; Jasinski *et al.*, 2011; Jasinski *et al.*, 2010*a*; Jasinski *et al.*, 2010*b*). In continuation of our studies of structural aspects of simple organic salts of amine bases, the title compound was synthesized.

Intracyclic C–C–C angles in the picrate anion markedly deviate from the ideal value by covering a range of 111.95 (12)–125.38 (13) ° where the smallest angle is found on the carbon atome bearing the deprotonated hydroxy group and the largest angle on one of the carbon atoms in *ortho* position to the former one. The cationic part demonstrates a relatively smaller distortion of its aromatic system in terms of intracyclic C–C–C angles, the latter ones found in between 118.25 (14) ° and 122.33 (13) °. The smallest angle in the cation appears on the unsubstituted carbon atom in between the protonated amine group and the chloro substituent while the largest angle is present on the carbon atom bearing the protonated amine group. The tilting of the nitro groups of the picrate anion with respect to the aromatic system they are bonded to varies significantly, the respective O–N–C–C dihedral angles being 13.5 (2) °, -25.4 (2) ° and 42.61 (19) °. The least-squares planes defined by the individual carbon atoms of both aromatic systems subtend an angle of 16.92 (7) ° (Fig. 1).

In the crystal, classical hydrogen bonds of the N–H···O type are observed, as well as C–H···O contacts whose range falls by more than 0.2 Å below the sum of van-der-Waals radii of the participating. atoms. The latter contacts are supported by carbon-bound hydrogen atoms on the cation as well as the anion and invariably have oxygen atoms on nitro groups as acceptors. Two of the nitrogen-bonded hydrogen atoms form hydrogen bonds to the oxygen atom of the deprotonated hydroxyl group while the third nitrogen-bonded hydrogen atom forms a hydrogen bond to a nitro group. The latter hydrogen bond shows bifurcation. Metrical parameters as well as information about the symmetry of these contacts are summarized in Table 1. In total, the N–H···O type hydrogen bonds connect the entities of the crystal structure to columnar arrays along the crystallographic *a* axis that are further connected to layers parallel *ab* by the C–H···O contacts. In terms of graph-set analysis (Etter *et al.*, 1990; Bernstein *et al.*, 1995), the descriptor for the C–H···O contacts is *DR*^2^_2_(10) on the unary level while the classical hydrogen bonds necessitate a *DDDDD* descriptor on the same level (Fig. 2).

The packing of the title compound in the crystal structure is shown in Figure 3.

### Experimental

3-Chloro-4-fluoroaniline (1.45 g, 0.01 mol) and picric acid (2.29 g, 0.01 mol) were individually dissolved in water (60 mL). The solutions were mixed and HCl (5 *M*, 2 mL) was added under stirring in a few minutes. The product formed was filtered and dried. Yellow crystals of the title compound were obtained by slow evaporation of a solution of the compound in ethanol at room temperature.

### Refinement

Carbon-bound H atoms were placed in calculated positions (C–H 0.95 Å) and were included in the refinement in the riding model approximation, with *U*(H) set to 1.2*U*_eq_(C). All nitrogen-bound H atoms were located on a difference Fourier map and refined freely.

### Crystal data

**Table:**

C_6_H_6_ClF^+^·C_6_H_2_N_3_O_7_^−^	*Z* = 2
*M**_r_* = 374.67	*F*(000) = 380
Triclinic, *P*1	*D*_x_ = 1.736 Mg m^−^^3^
Hall symbol: -P 1	Melting point: 438 K
*a* = 4.4054 (2) Å	Mo *K*α radiation, λ = 0.71073 Å
*b* = 11.9881 (5) Å	Cell parameters from 8065 reflections
*c* = 13.7010 (5) Å	θ = 2.3–28.3°
α = 90.057 (1)°	µ = 0.33 mm^−^^1^
β = 91.803 (1)°	*T* = 200 K
γ = 97.743 (1)°	Needle, yellow
*V* = 716.62 (5) Å^3^	0.53 × 0.32 × 0.13 mm

### Data collection

**Table:**

Bruker APEXII CCD diffractometer	3525 independent reflections
Radiation source: fine-focus sealed tube	2947 reflections with *I* > 2σ(*I*)
Graphite monochromator	*R*_int_ = 0.014
φ and ω scans	θ_max_ = 28.3°, θ_min_ = 2.3°
Absorption correction: multi-scan (*SADABS*; Bruker, 2008)	*h* = −5→5
*T*_min_ = 0.907, *T*_max_ = 1.000	*k* = −16→15
12360 measured reflections	*l* = −18→18

### Refinement

**Table:**

Refinement on *F*^2^	Primary atom site location: structure-invariant direct methods
Least-squares matrix: full	Secondary atom site location: difference Fourier map
*R*[*F*^2^ > 2σ(*F*^2^)] = 0.037	Hydrogen site location: inferred from neighbouring sites
*wR*(*F*^2^) = 0.097	H atoms treated by a mixture of independent and constrained refinement
*S* = 1.06	*w* = 1/[σ^2^(*F*_o_^2^) + (0.0377*P*)^2^ + 0.4436*P*] where *P* = (*F*_o_^2^ + 2*F*_c_^2^)/3
3525 reflections	(Δ/σ)_max_ < 0.001
238 parameters	Δρ_max_ = 0.32 e Å^−^^3^
0 restraints	Δρ_min_ = −0.27 e Å^−^^3^

### Fractional atomic coordinates and isotropic or equivalent isotropic displacement parameters (Å^2^)

**Table:**

	*x*	*y*	*z*	*U*_iso_*/*U*_eq_	
O1	0.6279 (2)	0.56237 (8)	0.18047 (8)	0.0272 (2)	
O11	0.1858 (3)	0.60505 (11)	0.04084 (9)	0.0397 (3)	
O12	0.4639 (3)	0.69531 (10)	−0.06804 (8)	0.0430 (3)	
O21	0.8395 (3)	1.07312 (9)	0.07332 (9)	0.0381 (3)	
O22	1.2124 (3)	1.06697 (10)	0.17928 (10)	0.0439 (3)	
O31	1.3048 (3)	0.72968 (13)	0.35325 (9)	0.0478 (3)	
O32	0.9194 (3)	0.59811 (10)	0.35634 (9)	0.0426 (3)	
N1	0.4035 (3)	0.67317 (10)	0.01741 (9)	0.0263 (3)	
N2	0.9866 (3)	1.02249 (11)	0.13271 (9)	0.0298 (3)	
N3	1.0560 (3)	0.68383 (11)	0.32143 (9)	0.0299 (3)	
C11	0.7050 (3)	0.66785 (12)	0.17296 (10)	0.0228 (3)	
C12	0.6014 (3)	0.73200 (12)	0.09313 (10)	0.0234 (3)	
C13	0.6869 (3)	0.84526 (12)	0.07879 (10)	0.0249 (3)	
H13	0.6095	0.8826	0.0242	0.030*	
C14	0.8896 (3)	0.90317 (12)	0.14653 (10)	0.0255 (3)	
C15	1.0104 (3)	0.84895 (12)	0.22500 (10)	0.0263 (3)	
H15	1.1546	0.8894	0.2695	0.032*	
C16	0.9185 (3)	0.73573 (12)	0.23741 (10)	0.0246 (3)	
Cl1	−0.28986 (12)	0.06232 (4)	0.38057 (4)	0.04913 (14)	
F1	0.1550 (3)	0.16950 (9)	0.52432 (7)	0.0473 (3)	
N4	0.0749 (3)	0.42503 (11)	0.19073 (9)	0.0252 (2)	
H41	−0.080 (5)	0.4671 (18)	0.1987 (15)	0.044 (6)*	
H42	0.026 (5)	0.3832 (17)	0.1376 (15)	0.037 (5)*	
H43	0.262 (5)	0.4739 (17)	0.1817 (14)	0.040 (5)*	
C21	0.0979 (3)	0.35612 (12)	0.27822 (10)	0.0240 (3)	
C22	−0.0891 (3)	0.25421 (12)	0.28441 (11)	0.0275 (3)	
H22	−0.2287	0.2282	0.2325	0.033*	
C23	−0.0676 (4)	0.19100 (12)	0.36846 (12)	0.0311 (3)	
C24	0.1372 (4)	0.23142 (14)	0.44313 (11)	0.0331 (3)	
C25	0.3238 (4)	0.33270 (14)	0.43629 (11)	0.0340 (3)	
H25	0.4635	0.3586	0.4882	0.041*	
C26	0.3048 (3)	0.39627 (13)	0.35244 (11)	0.0288 (3)	
H26	0.4318	0.4663	0.3460	0.035*	

### Atomic displacement parameters (Å^2^)

**Table:**

	*U*^11^	*U*^22^	*U*^33^	*U*^12^	*U*^13^	*U*^23^
O1	0.0247 (5)	0.0239 (5)	0.0325 (5)	0.0021 (4)	−0.0024 (4)	0.0049 (4)
O11	0.0309 (6)	0.0452 (7)	0.0386 (6)	−0.0094 (5)	−0.0049 (5)	0.0001 (5)
O12	0.0619 (8)	0.0383 (6)	0.0242 (5)	−0.0074 (6)	−0.0096 (5)	0.0047 (5)
O21	0.0448 (7)	0.0259 (5)	0.0441 (7)	0.0067 (5)	−0.0002 (5)	0.0062 (5)
O22	0.0430 (7)	0.0330 (6)	0.0513 (8)	−0.0089 (5)	−0.0068 (6)	−0.0017 (5)
O31	0.0333 (6)	0.0687 (9)	0.0396 (7)	0.0038 (6)	−0.0145 (5)	0.0077 (6)
O32	0.0586 (8)	0.0348 (6)	0.0334 (6)	0.0051 (6)	−0.0104 (5)	0.0090 (5)
N1	0.0279 (6)	0.0239 (6)	0.0266 (6)	0.0032 (5)	−0.0062 (5)	0.0012 (5)
N2	0.0325 (7)	0.0246 (6)	0.0319 (7)	0.0016 (5)	0.0041 (5)	−0.0013 (5)
N3	0.0312 (6)	0.0362 (7)	0.0238 (6)	0.0115 (5)	−0.0037 (5)	0.0000 (5)
C11	0.0205 (6)	0.0256 (6)	0.0227 (6)	0.0048 (5)	0.0011 (5)	0.0017 (5)
C12	0.0225 (6)	0.0252 (7)	0.0222 (6)	0.0026 (5)	−0.0018 (5)	−0.0011 (5)
C13	0.0260 (7)	0.0247 (7)	0.0244 (6)	0.0049 (5)	−0.0008 (5)	0.0020 (5)
C14	0.0264 (7)	0.0224 (6)	0.0276 (7)	0.0025 (5)	0.0015 (5)	0.0004 (5)
C15	0.0240 (7)	0.0304 (7)	0.0241 (7)	0.0028 (5)	−0.0008 (5)	−0.0031 (5)
C16	0.0228 (6)	0.0296 (7)	0.0217 (6)	0.0059 (5)	−0.0019 (5)	0.0019 (5)
Cl1	0.0631 (3)	0.0281 (2)	0.0538 (3)	−0.00226 (18)	−0.0007 (2)	0.01280 (18)
F1	0.0680 (7)	0.0454 (6)	0.0299 (5)	0.0140 (5)	−0.0025 (5)	0.0148 (4)
N4	0.0270 (6)	0.0236 (6)	0.0249 (6)	0.0038 (5)	−0.0040 (5)	0.0029 (5)
C21	0.0260 (7)	0.0241 (6)	0.0234 (6)	0.0089 (5)	−0.0004 (5)	0.0020 (5)
C22	0.0326 (7)	0.0225 (7)	0.0280 (7)	0.0071 (5)	−0.0031 (5)	0.0002 (5)
C23	0.0386 (8)	0.0222 (7)	0.0335 (8)	0.0077 (6)	0.0024 (6)	0.0044 (6)
C24	0.0442 (9)	0.0338 (8)	0.0239 (7)	0.0149 (7)	0.0017 (6)	0.0074 (6)
C25	0.0385 (8)	0.0394 (9)	0.0244 (7)	0.0078 (7)	−0.0057 (6)	0.0002 (6)
C26	0.0301 (7)	0.0291 (7)	0.0270 (7)	0.0037 (6)	−0.0028 (5)	0.0005 (6)

### Geometric parameters (Å, º)

**Table:**

O1—C11	1.2695 (17)	C15—H15	0.9500
O11—N1	1.2244 (16)	Cl1—C23	1.7234 (16)
O12—N1	1.2300 (17)	F1—C24	1.3439 (17)
O21—N2	1.2345 (17)	N4—C21	1.4651 (17)
O22—N2	1.2240 (18)	N4—H41	0.91 (2)
O31—N3	1.2253 (18)	N4—H42	0.89 (2)
O32—N3	1.2244 (18)	N4—H43	0.96 (2)
N1—C12	1.4524 (17)	C21—C22	1.383 (2)
N2—C14	1.4510 (18)	C21—C26	1.385 (2)
N3—C16	1.4623 (17)	C22—C23	1.387 (2)
C11—C12	1.4355 (19)	C22—H22	0.9500
C11—C16	1.4375 (19)	C23—C24	1.385 (2)
C12—C13	1.3754 (19)	C24—C25	1.377 (2)
C13—C14	1.386 (2)	C25—C26	1.386 (2)
C13—H13	0.9500	C25—H25	0.9500
C14—C15	1.386 (2)	C26—H26	0.9500
C15—C16	1.376 (2)		
			
O11—N1—O12	123.14 (12)	C11—C16—N3	119.69 (12)
O11—N1—C12	119.24 (12)	C21—N4—H41	108.0 (13)
O12—N1—C12	117.62 (12)	C21—N4—H42	112.0 (13)
O22—N2—O21	123.61 (13)	H41—N4—H42	106.5 (18)
O22—N2—C14	118.27 (13)	C21—N4—H43	110.8 (12)
O21—N2—C14	118.10 (13)	H41—N4—H43	109.2 (18)
O32—N3—O31	123.48 (13)	H42—N4—H43	110.1 (17)
O32—N3—C16	119.18 (13)	C22—C21—C26	122.33 (13)
O31—N3—C16	117.33 (13)	C22—C21—N4	119.08 (12)
O1—C11—C12	122.68 (12)	C26—C21—N4	118.59 (13)
O1—C11—C16	125.23 (12)	C21—C22—C23	118.25 (14)
C12—C11—C16	111.95 (12)	C21—C22—H22	120.9
C13—C12—C11	125.38 (13)	C23—C22—H22	120.9
C13—C12—N1	116.10 (12)	C24—C23—C22	119.53 (14)
C11—C12—N1	118.42 (12)	C24—C23—Cl1	119.67 (12)
C12—C13—C14	117.90 (13)	C22—C23—Cl1	120.80 (12)
C12—C13—H13	121.1	F1—C24—C25	119.18 (15)
C14—C13—H13	121.1	F1—C24—C23	118.91 (15)
C13—C14—C15	121.54 (13)	C25—C24—C23	121.91 (14)
C13—C14—N2	119.12 (13)	C24—C25—C26	118.99 (14)
C15—C14—N2	119.29 (13)	C24—C25—H25	120.5
C16—C15—C14	118.98 (13)	C26—C25—H25	120.5
C16—C15—H15	120.5	C21—C26—C25	118.99 (14)
C14—C15—H15	120.5	C21—C26—H26	120.5
C15—C16—C11	124.21 (12)	C25—C26—H26	120.5
C15—C16—N3	116.09 (12)		
			
O1—C11—C12—C13	−177.10 (13)	C12—C11—C16—C15	0.7 (2)
C16—C11—C12—C13	−1.1 (2)	O1—C11—C16—N3	−2.3 (2)
O1—C11—C12—N1	−0.8 (2)	C12—C11—C16—N3	−178.15 (12)
C16—C11—C12—N1	175.13 (12)	O32—N3—C16—C15	155.61 (14)
O11—N1—C12—C13	−137.00 (14)	O31—N3—C16—C15	−24.3 (2)
O12—N1—C12—C13	42.61 (19)	O32—N3—C16—C11	−25.4 (2)
O11—N1—C12—C11	46.39 (19)	O31—N3—C16—C11	154.69 (14)
O12—N1—C12—C11	−134.00 (14)	C26—C21—C22—C23	0.3 (2)
C11—C12—C13—C14	−0.1 (2)	N4—C21—C22—C23	−178.97 (13)
N1—C12—C13—C14	−176.40 (13)	C21—C22—C23—C24	0.3 (2)
C12—C13—C14—C15	1.8 (2)	C21—C22—C23—Cl1	−179.12 (11)
C12—C13—C14—N2	179.32 (13)	C22—C23—C24—F1	179.76 (14)
O22—N2—C14—C13	−164.06 (14)	Cl1—C23—C24—F1	−0.8 (2)
O21—N2—C14—C13	14.7 (2)	C22—C23—C24—C25	−0.6 (2)
O22—N2—C14—C15	13.5 (2)	Cl1—C23—C24—C25	178.80 (13)
O21—N2—C14—C15	−167.71 (14)	F1—C24—C25—C26	180.00 (15)
C13—C14—C15—C16	−2.2 (2)	C23—C24—C25—C26	0.4 (3)
N2—C14—C15—C16	−179.71 (13)	C22—C21—C26—C25	−0.5 (2)
C14—C15—C16—C11	0.9 (2)	N4—C21—C26—C25	178.73 (14)
C14—C15—C16—N3	179.77 (13)	C24—C25—C26—C21	0.2 (2)
O1—C11—C16—C15	176.55 (13)		

### Hydrogen-bond geometry (Å, º)

**Table:**

*D*—H···*A*	*D*—H	H···*A*	*D*···*A*	*D*—H···*A*
N4—H41···O1^i^	0.91 (2)	1.85 (2)	2.7324 (17)	165 (2)
N4—H42···O12^ii^	0.89 (2)	2.40 (2)	3.0599 (18)	131.2 (16)
N4—H42···O11^ii^	0.89 (2)	2.60 (2)	3.3425 (17)	141.8 (16)
N4—H43···O1	0.96 (2)	1.81 (2)	2.7579 (16)	172.7 (18)
C13—H13···O21^iii^	0.95	2.47	3.3152 (19)	148
C26—H26···O32	0.95	2.49	3.378 (2)	156

Symmetry codes: (i) *x*−1, *y*, *z*; (ii) −*x*, −*y*+1, −*z*; (iii) −*x*+1, −*y*+2, −*z*.
